# Sympathetic Ophthalmitis

**Published:** 1892-06-04

**Authors:** 


### OPHTHALMOLOGY.

### SYMPATHETIC OPHTHALMITIS.

Sympathetic ophthalmitis is the inflammation which occurs in the fellow eye after injury of the other eye. It makes its appearance any time after three weeks have elapsed from the date of the injury, and is a most destructive process, although in mild cases recovery is possible. Perhaps from two to three months from the time of injury of the other eye, may be stated as the most common date of the onset of the symptoms, but when the injured eye contains a foreign body, the sympathetic ophthalmitis may not occur for a year or more, in fact, not until something disturbs the foreign body, which heretofore has been lying in a quiescent state.

Therefore, in this disease we always have a history of injury to the other eye, at some period more remote than three weeks from the date of consultation, and preceded for some days by more or less inflammation of the injured eye.

The sympathising eye is irritable and weak, there is photophobia, and injection and lachrymation when exposed to the light, accommodation is impaired, and therefore near vision is troublesome, and the eye cannot be used for it for any length of time, the patient complaining of cloudiness. There is severe pain in the eye and temple, the symptoms getting worse till the patient complains of darkness, or passing off for a while, to recur later on. It affects chiefly the uveal tract, with inflammation of the iris, ciliary body, and choroid. Deposits are formed on the back of the cornea (keratitis punctata), and the iritis being of a plastic nature, posterior synechiæ form, till the iris is completely tied down to the anterior capsule of the lens; the iris is muddy in colour, and shows numerous blood vessels, the lens may become cataractous and eventually absorbed, the vitreous is often affected, and sometimes there is also neuro-retinitis. The prognosis is exceedingly grave, but if the excitant eye be removed early it seems to be much more favourable. Out of 64 cases in which the exciting eye was removed within a short time of the onset of sympathetic inflammation, in only eight cases was the sympathising eye known to be lost.* In 65 the exciter was either not removed at all, or not till long after the sympathetic disease had set in, and in no less than 26 of these the sympathiser was lost.

From these figures we can but conclude that at any rate removal does not increase the danger to the second eye, while it probably renders a better chance of escape, but the number of cases reported on, only two hundred, is not enough to hazard any definite opinion. As regards operations on the sympathising eye, the Committee reported on a very small number in which iridectomy was performed (eleven cases), the eye operated on was completely lost in three of these, only one recovered completely, and that was a doubtful case, the remaining seven retained a certain amount of vision.

The general opinion seems to be that when sympathetic ophthalmitis has once set in, little can be done, the exciting eye should not be interfered with, unless disorganised, as after the attack it may be the better of the two, and that iridectomy in the sympathising eye will probably be unsuccessful. Application of atropine should be made, rest from light in a dark room, leeches, blisters, and setons, are all to be used, whilst the exhibition of mercury is by some considered a *sine qua non*, and by others unnecessary.

* Report of Committee on Sympathetic Ophthalmitis to the Ophthalmological Society.

